# Characterizing the photoluminescence of fluorescein-labeled cellulose in aqueous and alcohol solutions: influence of the cellulose backbone

**DOI:** 10.1038/s41598-024-72773-6

**Published:** 2024-10-31

**Authors:** Chi-Yang Yen, Shailesh Rana, Kamlesh Awasthi, Nobuhiro Ohta, Masahito Oh-e

**Affiliations:** 1 Institute of Photonics Technologies, Department of Electrical Engineering, National Tsing Hua University, 101 Sec. 2 Kuang-Fu Road, Hsinchu, 300044 Taiwan; 2 Department of Applied Chemistry, Institute of Molecular Science, Center for Emergent Functional Matter Science, National Yang Ming Chiao Tung University, 1001 Ta-Hsueh Road, Hsinchu, 300093 Taiwan

**Keywords:** Cellulose, Fluorescein, Photoluminescence, Fluorescence, Quenching, Lifetime, Photochemistry, Physical chemistry, Biomaterials, Condensed-matter physics, Soft materials, Fluorescent probes, Chemical physics

## Abstract

Although many dyes have been introduced into cellulose, whether bound to its backbone or within a cellulose matrix, few studies have determined whether the backbone statically or dynamically quenches the photoluminescence of the dye. To advance cellulosic fluorescent films, the influence of the cellulose backbone on photoluminescence must be understood. We determined the fluorescence properties of fluorescein isothiocyanate (FITC) and fluorescein-labeled cellulose (FLC) in water and alcohol, including their quantum yields $\boldsymbol{\phi}_{\textit{\textbf{PL}}}$, lifetimes ${\boldsymbol{\tau}}$, and rates of radiative ${\textit{\textbf{k}}}_{\textit{\textbf{r}}}$ and nonradiative ${\textit{\textbf{k}}}_{\textit{\textbf{nr}}}$ decay. Dissolved FLC had a ~ 30× lower $\boldsymbol{\phi}_{\textit{\textbf{PL}}}$ than FITC, suggesting that incorporating FITC into the cellulose backbone remarkably reduces the fluorescence efficiency. The FLC solutions had a six-fold lower ${\textit{\textbf{k}}}_{\textit{\textbf{r}}}$ than their FITC counterparts but a 10–20 times higher ${\textit{\textbf{k}}}_{\textit{\textbf{nr}}}$. Presumably, this was because the cellulose backbone interacted weakly with the fluorescein moieties, suggesting a quenching mechanism that can be termed quasi-static, corresponding to static quenching between the fluorescein moieties and cellulose backbone, in addition to the fluorescence quenching caused by the intramolecular nonradiative processes of fluorescein, as observed in conventional molecules. Using the Strickler‒Berg formula, we deduced the analytical radiative decay rate constants ${\textit{\textbf{k}}}_{\textit{\textbf{r}}}^{\textit{\textbf{S.B.}}}$ and eventually estimated the number of very short-lived fluorescein moieties per single fluorescent fluorescein moiety, corresponding well with static quenching.

**Supplementary Information:**

The online version contains supplementary material available at 10.1038/s41598-024-72773-6.

## Introduction

Cellulose science and technology have been actively advancing in a wide range of disciplines, including chemistry, biology, physics, and photonics. Cellulose is an abundant renewable biomaterial produced by plants via biosynthesis, forming microfibrils that further assemble into cellulose fibers^1–3^. In addition to the eco-friendly, sustainable nature of cellulose, various useful properties have been unveiled such as high strength^4^, light weight^5^, low thermal expansion^6^, and other unique properties^7–13^.

Photonics researchers are interested in how the iridescence of cellulose films can be induced by the alignment and helical pitch of cellulose nanocrystals. Such coloration is observed on the external surfaces of living creatures^14–22^; however, unlike natural processes, controlling the order of molecular scales from nanoscopic to macroscopic lengths remains challenging^23–27^. A recent optoelectronic application of cellulose nanocrystals is cellulose pigments^28^, which have also been investigated for large-scale fabrication^29^.

Integrating functionality into sustainably sourced fluorescent films using renewable resources is being actively investigated^30–32^; however, many conventional fluorescent films are made from unsustainable resources that face technical economic issues in recycling, greatly impacting ecosystems. From this perspective, fluorescent films should preferably be developed from renewable resources while merging functionality with sustainability. Of particular interest are cellulose films incorporated with fluorescent dyes, as these films could perfectly combine the inherent functionalities of the dyes with the excellent optical and mechanical properties of these naturally sourced films^33–37^.

To develop such fluorescent films from cellulose, however, we must carefully pursue a comprehensive understanding of the host/guest interactions between cellulose and fluorescent molecules. The microenvironmental rigidity of polymers including cellulose is well known^38–43^; when dyes are linked to cellulose, the microenvironmental (i.e., local) rigidity may increase, which usually decreases the rate of nonradiative decay and thus increases the quantum yield. Fluorescein is a very common, highly fluorescent dye that is used as a tracer in a wide range of applications, including forensics, analytical chemistry, and biomedical analysis^44–47^. Despite its popularity, however, it is typically used with unsustainable substrates, which raises the questions of how sustainable cellulose as a medium interacts with fluorescein and how the presence of the cellulose backbone affects its photoluminescence (PL) properties. The PL properties of this popular dye have been thoroughly studied^48–51^, including the effects of solvent and temperature^52–57^ in the ground and excited states. Despite the increasing use of cellulose as a medium, however, few studies have examined how cellulose interacts with fluorescein, although the fluorescence of a fluorescein derivative was reportedly enhanced on the cellulose backbone^58^. Moreover, the microenvironmental rigidity of the cellulose backbone has been reported to increase the quantum yield and decrease the non-radiative decay^38–43^, but we have observed the opposite effect on the quantum yield of fluorescein. Given that the literature on this specific topic is limited, we sought to more comprehensively answer the fundamental question of how the cellulose backbone affects the PL properties of dyes.

**Fig. 1:**
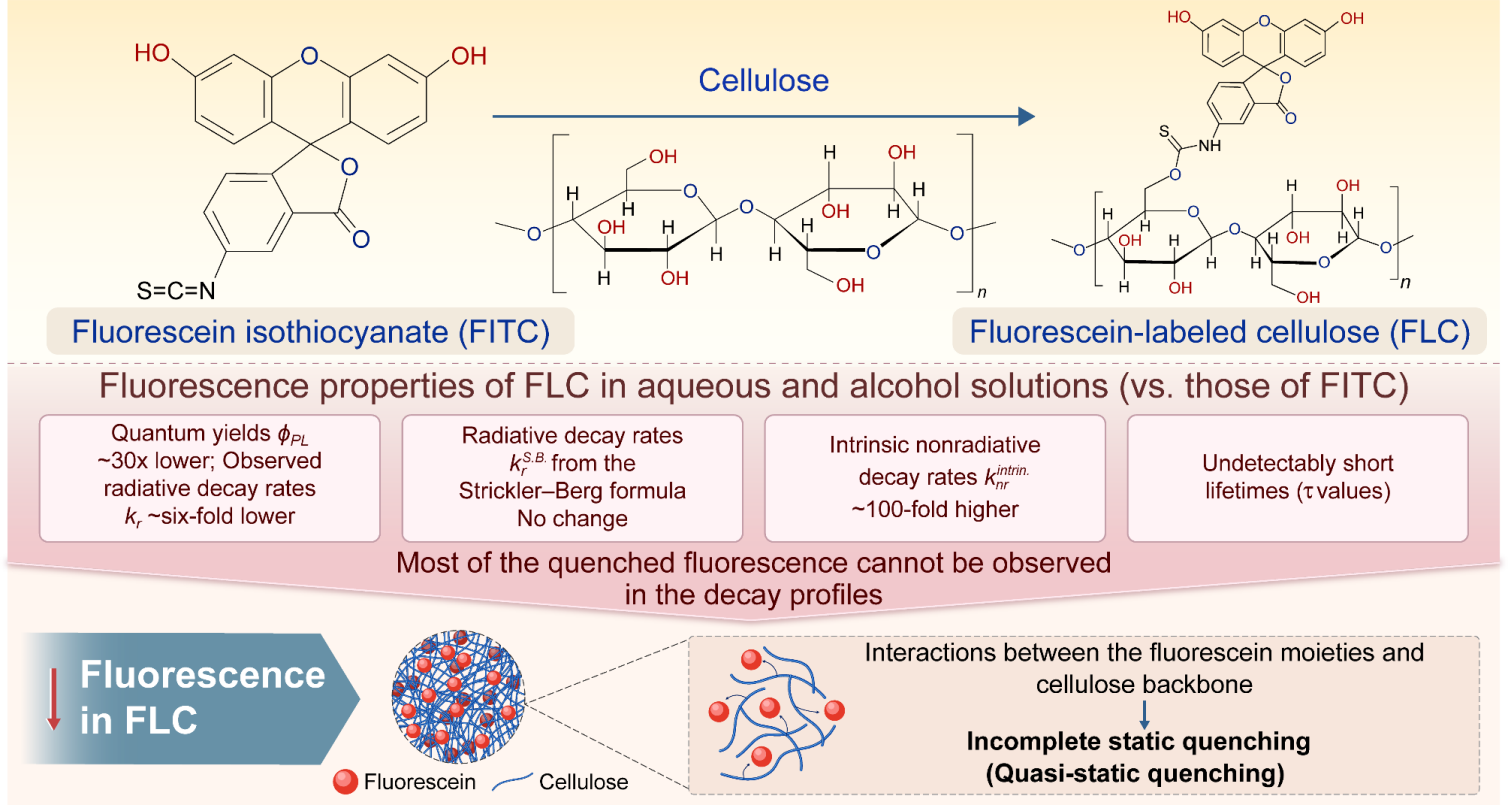
Graphical depiction of the investigation, including the chemical structures of the materials being compared and a summary of the results.

Accordingly, we aimed to compare the PL properties of fluorescein with and without a cellulose backbone to fundamentally understand how the cellulose backbone influences the fluorescence of dye molecules. In particular, we compared the PL properties of fluorescein isothiocyanate (FITC)^59^ with those of fluorescein-labeled cellulose (FLC) in aqueous and alcoholic solutions. Figure 1 provides an overview of the investigation, along with schematics of the materials compared and a summary of the results discussed herein. Observed differences in their fluorescence behaviors revealed how the interactions between the cellulose backbone and fluorescein moieties affect the PL properties of fluorescein. Our intention is not necessarily to demonstrate the use of fluorescein for practical fluorescent film applications; however, dyes are being introduced into cellulose chains or matrices without serious consideration of the photophysics of the dyes. Nonetheless, although some reports have been published from the viewpoint of the microenvironmental rigidity of the cellulose backbone^38–41,43^, to the best of our knowledge, little has been discussed about how the cellulose backbone quenches PL. This study reveals the existence of a quenching mechanism and highlights the need for caution when fluorescent dyes will be bound to the cellulose backbone.

## Results and discussion

**Fig. 2:**
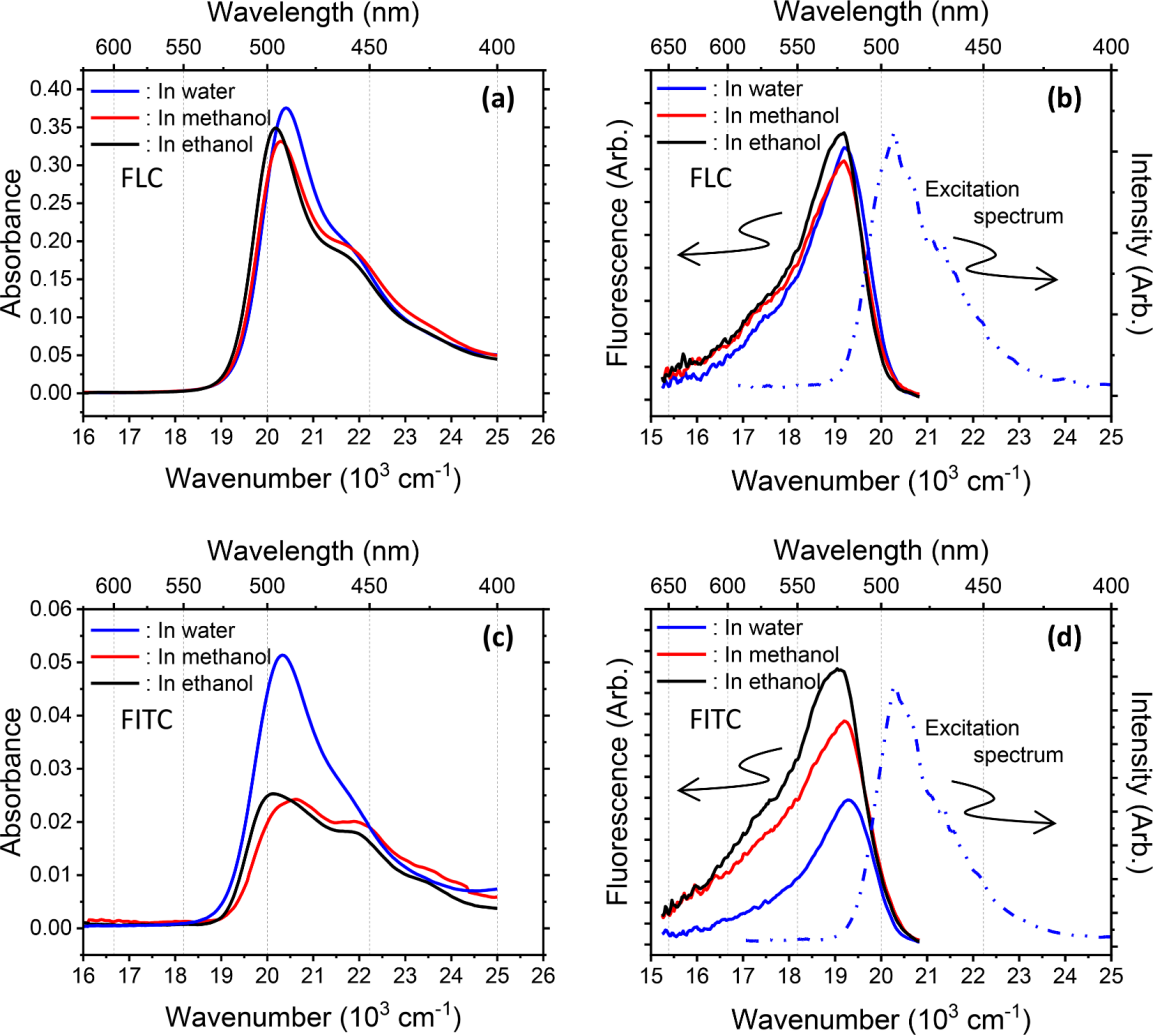
(**a**) UV‒Vis absorption and (**b**) fluorescence spectra of FLC solutions with ${C}_{FITC}^{FLC}=7.31\:{\upmu}\text{M}$. Note that ${C}_{FITC}^{FLC}$ is calculated using 7.55 fluorescein moieties per single chain. The number of fluorescein moieties is discussed in “Quantifying the fluorescein moieties per chain.” (**c**) (**d**) Corresponding FITC solutions with ${C}_{FITC}=1.00\:{\upmu}\text{M}$. The excitation spectra in (**b**) and (**d**) were recorded from aqueous solutions, and those from other solutions also matched the absorption spectra well. The excitation wavelength for recording the fluorescence spectra was 450 nm.

Solid states experience various deactivation processes from photoexcited states, such as excitation energy transfer, exciton delocalization, the trapping of energy transfer and excitons, and the formation of a nonradiative aggregate in an excited state^60–62^. To avoid such complex processes and probe only the interactions between the fluorescein moieties and cellulose backbone, we investigated diluted solutions instead of films. Figure 2 compares the ultraviolet‒visible (UV‒Vis) absorption and fluorescence spectra with representative excitation spectra of the FLC and FITC solutions. In the absorption spectra of the FLC solutions, changing the solvent scarcely affects the absorption properties, except for slight shifts in the maximum peak wavelength (${\lambda}_{max}$), which indicates the electronic transition from the ground to the first excited state (S_0_ → S_1_). This transition is attributed mainly to the transition from the highest-occupied molecular orbital (HOMO) to the lowest-occupied molecular orbital (LUMO) with a small contribution from HOMO-5 → LUMO^59,63,64^. The HOMO and LUMO are known to be located on the xanthene moiety^59,63^.

The ${\lambda}_{max}$ absorbances of FLC are 490, 493, and 495 nm (all with a shoulder near 470 nm) in water, methanol, and ethanol, respectively, which have dielectric constants of 78.3, 32.7, and 24.6 at 25 °C. Although we avoided considering shifts in ${\lambda}_{max}$ owing to the few available solvents and the lack of clear variations beyond the error range, the polarity property of a solvent generally influences ${\lambda}_{max}$, which could also possibly be affected by the different structures caused by different solvents. However, we recognize that the absorption spectra of the FLC solutions changed much less than those of their FITC counterparts. In the absorption spectra of FITC in water, methanol, and ethanol, the ${\lambda}_{max}$ values of the solutions are 492, 487, and 497 nm, respectively, which are shifted only slightly from those of their FLC counterparts. Surprisingly, however, the type of solvent significantly influences the shape of the absorbance spectra, and the absorbance at ${\lambda}_{max}$ is significantly higher in water than that in the alcohols (Fig. 2(c)).

The solvent also affected the fluorescence spectra of the FLC and FITC solutions, as shown in Fig. 2(b) and (d). The effect on FLC was far smaller than that on FITC, however, with the fluorescence intensity of the latter varying significantly. The excitation spectra of each sample agreed well with the absorption spectra; thus, the fluorescence originated from FITC itself and the fluorescein moieties of the FLC.

Notable solvent dependence was observed in the absorption and fluorescence spectra of FITC, whereas those of FLC scarcely changed, except for the slight shifts in the peaks. As these spectra were recorded to probe the fluorescein moieties, the distinctions between the spectral features of FLC and FITC must depend on whether cellulose backbones are present. From this perspective, the cellulose backbones surround and interact with their fluorescein moieties as well as those of neighboring molecules, thus shielding them from the solvent environment.

On the other hand, FITC is sensitive to the potential of hydrogen (i.e., the pH) and forms multiple species, including some tautomers^51,65^. The multiple prototropic forms of FITC are due to the presence of its xanthene and benzoic acid moieties, which can exist in multiple ionization states^59,66^. The benzoate carboxyl group also enables lactone formation. However, the pH of both the alcohol and aqueous solutions in our system was within the range of 6.2‒6.9. Further, the fluorescence spectra were acquired at 450 nm excitation, but we confirmed that the fluorescence spectra recorded with excitation at 490 nm were the same as those excited at 450 nm. The pH dependence would be expected to cause a large difference in the fluorescence spectra between excitation at 450 and 490 nm; however, this was not the case with our system.

**Fig. 3:**
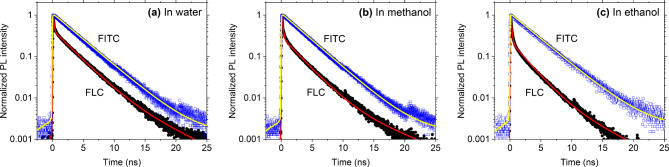
Semi-log plot of the normalized PL decay profiles of FLC and FITC in (**a**) water, (**b**) methanol, and (**c**) ethanol. The black and blue dots in the graphs are experimental values for FLC and FITC, respectively, while the solid red and yellow lines are the corresponding analytical fits.

To reveal the interactions between the fluorescein moieties and cellulose backbone, we acquired the fluorescence properties of the FLC and FITC solutions, including the PL quantum yields ${\phi}_{PL}$, lifetimes ${\tau}$, and rate constants ${k}_{r}$ and ${k}_{nr}$ of radiative and nonradiative decay, respectively. The ${\phi}_{PL}$ of each sample was quantified using quinine sulfate (${\phi}_{R}=0.54$) in 0.1 N H_2_SO_4_ solution and fluorescein sodium salt (FSS) in 0.1 N NaOH solution as a fluorescence standard. The ${\phi}_{PL}$ of the latter determined using that of the former with excitation at 350 nm was approximately 0.92, which is almost the same as the reference value^67^, demonstrating the precision of the method. FSS (${\phi}_{R}=0.92$) in 0.1 N NaOH solution with excitation at 450 nm was then used to determine the ${\phi}_{PL}$ of other samples. Experimentally, ${k}_{r}$ and ${k}_{nr}$ can be deduced from ${\tau}$, which must be determined by carefully fitting the fluorescence decay profiles recorded using time-correlated single photon counting. Figure 3 shows the typical PL decay profiles of each sample. Table 1 summarizes the ${\tau}$ values obtained for each solution. The decay profiles were fitted as a mono-exponential decay, i.e., ${A}_{1}\text{exp}\left(-t/{\tau}_{1}\right)$, or a bi-exponential decay, i.e., ${A}_{1}\text{exp}\left(-t/{\tau}_{1}\right)+{A}_{2}\text{exp}\left(-t/{\tau}_{2}\right)$, from which the lifetimes ${\tau}_{i}$ and pre-exponential factors ${A}_{i}$ were calculated together with the average lifetime ${\tau}_{ave}$. The PL decay profiles of FITC that we acquired are mono-exponential, from which the single component ${\tau}_{1}$ values in each solution were obtained, as listed in Table 1. These findings are in agreement with previously reported ones^68,69^, which corroborates our new observations on the PL decay profiles of the FLC solutions, which exhibit a bi-exponential function with two components, a fast and slow decay.

**Table 1:** PL lifetimes ${\tau}_{i}$ deduced from the fluorescence decay profiles of FLC and FITC in water, methanol, and ethanol.

Solute	Solvent	$\:{\tau}_{1}\:/\:ns\:({A}_{1})$	$\:{\tau}_{2}\:/\:ns\:({A}_{2})$	$\:{\tau}_{avg}\:/\:ns$
FLC	Water	0.09 (0.77)	2.79 (0.23)	0.71
	Methanol	0.12 (0.82)	2.69 (0.18)	0.58
	Ethanol	0.13 (0.86)	2.46 (0.14)	0.46
FITC	Water	3.27 (1.00)	-	3.27
	Methanol	3.25 (1.00)	-	3.25
	Ethanol	3.53 (1.00)	-	3.53

The fluorescence was monitored at λ = 520 nm. ${\tau}_{1}$, ${A}_{1}$, ${\tau}_{2}$, and ${A}_{2}$ are the parameters for fitting the PL decay profiles using ${A}_{1}\text{exp}\left(-t/{\tau}_{1}\right)+{A}_{2}\text{exp}\left(-t/{\tau}_{2}\right)$. The average lifetime (${\tau}_{avg}$) was estimated by $\left({A}_{1}{\tau}_{1}+{A}_{2}{\tau}_{2}\right)/\left({A}_{1}+{A}_{2}\right)$.

Table 2 summarizes the experimentally obtained ${\phi}_{PL}$, ${k}_{r}$, and ${k}_{nr}$, together with the analytic ${k}_{r}^{S.B.}$ calculated for each solution using the Strickler‒Berg formula^70^. In this analysis, ${k}_{r}$ and ${k}_{nr}$ were determined by ${\phi}_{PL}/{\tau}_{avg}$ and $1/{\tau}_{avg}-{k}_{r}$, respectively. To estimate the ${k}_{r}^{S.B.}$ of FLC, the number of fluorescein moieties incorporated into a single cellulose chain was considered to be in the range of ~ 7–14, as described in “Quantifying the fluorescein moieties per chain.” Further, each fluorescein moiety in the FLC solutions was assumed to be identical to that in the aqueous FITC solutions, as the absorption spectra of the two types of solutions are very similar. The ${\phi}_{PL}$ values of FITC that we acquired are close to previous values obtained at roughly pH 6.5^71^. Significantly, however, the ${\phi}_{PL}$ of FLC in each solution was one order of magnitude lower than that of its FITC counterpart. This experimentally observed reduction in ${\phi}_{PL}$ together with the shielding effect observed in the absorption and fluorescence spectra suggest interactions between the fluorescein moieties and cellulose backbone in FLC. The cellulose backbone does not exhibit absorption from 400 to 550 nm; thus, fluorescence resonance energy transfer does not occur. The microenvironmental rigidity created by polymer chains is often discussed to determine how dye molecules interact with polymers^38–41,43^. If this property of the cellulose backbone influences the PL behavior of fluorescein molecules, the nonradiative decay rates should decrease; however, we observed the opposite. Presumably, a different kind of interaction between the fluorescein moieties and cellulose backbone would play a role in causing the opposite behavior.

**Table 2:** PL properties of FLC and FITC.

Solute	Solvent	$\:{\phi}_{PL}$	$\:{k}_r \times\:{10}^{8}\:/\:{s}^{-1}$	$\:{k}_{nr} \times\:{10}^{9}\:/\:{s}^{-1}$	$\:{k}_r^{S.B.}\,\times\:{10}^{8}\:/\:{s}^{-1}$
FLC	Water	0.014	0.19	1.39	1.40‒2.53^*^
	Methanol	0.014	0.23	1.69	1.36‒2.45^*^
	Ethanol	0.018	0.38	2.14	1.41‒2.54^*^
FITC	Water	0.40	1.22	0.18	2.35
	Methanol	0.52	1.60	0.15	1.47
	Ethanol	0.67	1.90	0.09	1.70

The fluorescence was monitored at λ = 520 nm. ${\phi}_{PL}$, ${k}_{r}$, and ${k}_{nr}$ are the quantum yield and the rate constants of radiative and nonradiative decay, respectively. ${k}_{r}^{S.B.}$ is the rate constant of radiative decay calculated using the Strickler‒Berg formula. ^*^See the text for the estimation of these values.

As with FITC, the excitation and absorption spectra of each FLC solution were the same; thus, it is only the fluorescein moieties of FLC that fluoresce. Surprisingly, however, each FLC solution yielded an unusually low ${k}_{r}$, which was approximately six-fold lower than that of its FITC counterpart and roughly 10-fold lower than analytically estimated ${k}_{r}^{S.B.}$. More specifically, not only was the ${k}_{r}$ derived from ${\phi}_{PL}/{\tau}_{avg}$ approximately 10-fold lower than ${k}_{r}^{S.B.}$, but so was the ${k}_{r}^{fast}$ obtained by dividing the quantum yield of the fast component ${\phi}_{PL}^{fast}$ by the lifetime of the fast component ${\tau}_{1}$, suggesting that most of the quenched fluorescence cannot be observed in the decay profiles. The fast ${\tau}_{1}$ and slow ${\tau}_{2}$ components decomposed from the PL decay profile of FLC are summarized in the Supplementary Information (Supplementary Tables S1 and S2). Attributing the decrease in ${\phi}_{PL}$ to the decrease in ${k}_{r}$, however, would hardly be reasonable, because ${k}_{r}$ tends not to change when the fluorophore has the same extinction coefficient^72^, as is the case in our systems, considering that the fluorophores of FLC are still fluorescein moieties. Further, the increase in ${k}_{nr}$ suggests that the strong quenching must be due to static quenching.

Although in typical static quenching, PL is completely quenched by statically formed complexes and species^73,74^, we assumed that weak interactions occur between the fluorescein moieties and cellulose backbone, which enable some but not complete quenching. In addition, we considered the case wherein the statically but incompletely quenched PL is too fast to be detected under our experimental conditions, which can be termed quasi-static quenching.

Based on this model, we estimated the intrinsic ${k}_{nr}$ values $\left({k}_{nr}^{intrin.}\right)$ of FLC with ${\phi}_{PL}$ and the rate constants ${k}_{r}^{S.B.}$ for radiative decay calculated using the Strickler‒Berg formula^70^. Table 2 reveals that the ${k}_{r}^{S.B.}$ values of FITC are on the same order of magnitude as the corresponding ${k}_{r}$ values; therefore, it is reasonable to assume that the ${k}_{r}^{S.B.}$ values of FITC are equivalent to those of the fluorescein moieties of FLC, because FITC is intrinsically the same fluorophore as that of FLC. In fact, the calculated ${k}_{r}^{S.B.}$ values for FLC were almost identical to those for FITC.

### Quantifying the fluorescein moieties per chain

Note that there is ambiguity in quantifying the concentration ${C}_{FITC}^{FLC}$ of the fluorescein moieties in each FLC solution, which depends on the number of fluorescein moieties incorporated into a single cellulose backbone. As the specification for the custom degree of substitution, approximately 14 fluorescein moieties were designed for incorporation into a single cellulose backbone. According to our investigation, however, a single cellulose chain contained seven to eight fluorescein moieties, deviating approximately two-fold from the product specification. This fact was deduced by comparing the absorption spectra of the FLC and FITC solutions, because the ratio between their respective FITC concentrations should be equal to the ratio between their absorbances. This possible discrepancy in the number of fluorescein moieties in a single chain prompted us to consider a range of ${C}_{FITC}^{FLC}$ rather than a single value. Dye extinction coefficients often vary with the solvent, and analogously, fluorescein moieties bound to the cellulose backbone do not necessarily exhibit the same extinction coefficients as unbound FITC molecules, given the difference in their surrounding media or environments. From these perspectives, that is also why it is reasonable to consider a range of ${C}_{FITC}^{FLC}$ values rather than a single value. The range of the ${k}_{r}^{S.B.}$ of the FLC solutions given in Table 2 arises from this ambiguity. In typical static quenching, the absorption spectral features change as a function of the quencher concentration. This ambiguity, however, prevented us from accurately determining differences in the absorption spectra of FLC and FITC, including their absorption coefficients. Rather, weak interactions may be the ones that do not change the absorption spectrum.

According to the absorbance ratio, ${C}_{FITC}^{FLC}$ was quantified to be 7.31‒13.2 µM, which corresponds to 7.55‒14.0 fluorescein moieties per single chain. Accordingly, the ${k}_{r}^{S.B.}$ values in the FLC solutions were calculated, as summarized in Table 2, and they were indeed on the same order of magnitude as ${k}_{r}^{S.B.}$ and ${k}_{r}$ in the FITC solutions. From the ${\phi}_{PL}$ and ${k}_{r}^{S.B.}$ values in water, methanol, and ethanol, ${k}_{nr}^{intrin.}$ for each solution can eventually be deduced using ${k}_{nr}^{intrin.}={k}_{r}^{S.B.}(1-{\phi}_{PL})/{\phi}_{PL}$, i.e., ${k}_{nr}^{intrin.}=\:$1.02‒1.83, 0.994‒1.79, and 0.792‒1.43 × 10^10^ s^–1^, respectively, with corresponding values of ${\tau}^{intrin.}=1/({k}_{r}^{S.B.}+{k}_{nr}^{intrin.})=\:$53.8‒97.1, 55.1‒99.3, and 68.9‒124 ps. These time scales are obviously different from the ${\tau}_{avg}$ deduced from the PL decay profiles of FLC summarized in Table 1, suggesting the existence of an unexpected fast PL decay component that could not be identified in the present experiments. Note that the PL lifetime of an extremely short-lived component (${\tau}_{s}$) cannot be quantified, as our system does not allow distinguishing speeds below 60 ps. Therefore, we estimate possible time scales for an extremely fast component together with the pre-exponential factor by dividing the ${\tau}^{intrin.}$ values obtained from ${k}_{r}^{S.B.}$ into the experimentally observed ${\tau}_{i}$ and the undetectably short-lived component ${\tau}_{s}$, assuming that the PL decay profiles can be given by ${A}_{s}\text{exp}(-t/{\tau}_{s})+{A}_{1}\text{exp}(-t/{\tau}_{1})+{A}_{2}\text{exp}(-t/{\tau}_{2})$.

**Fig. 4:**
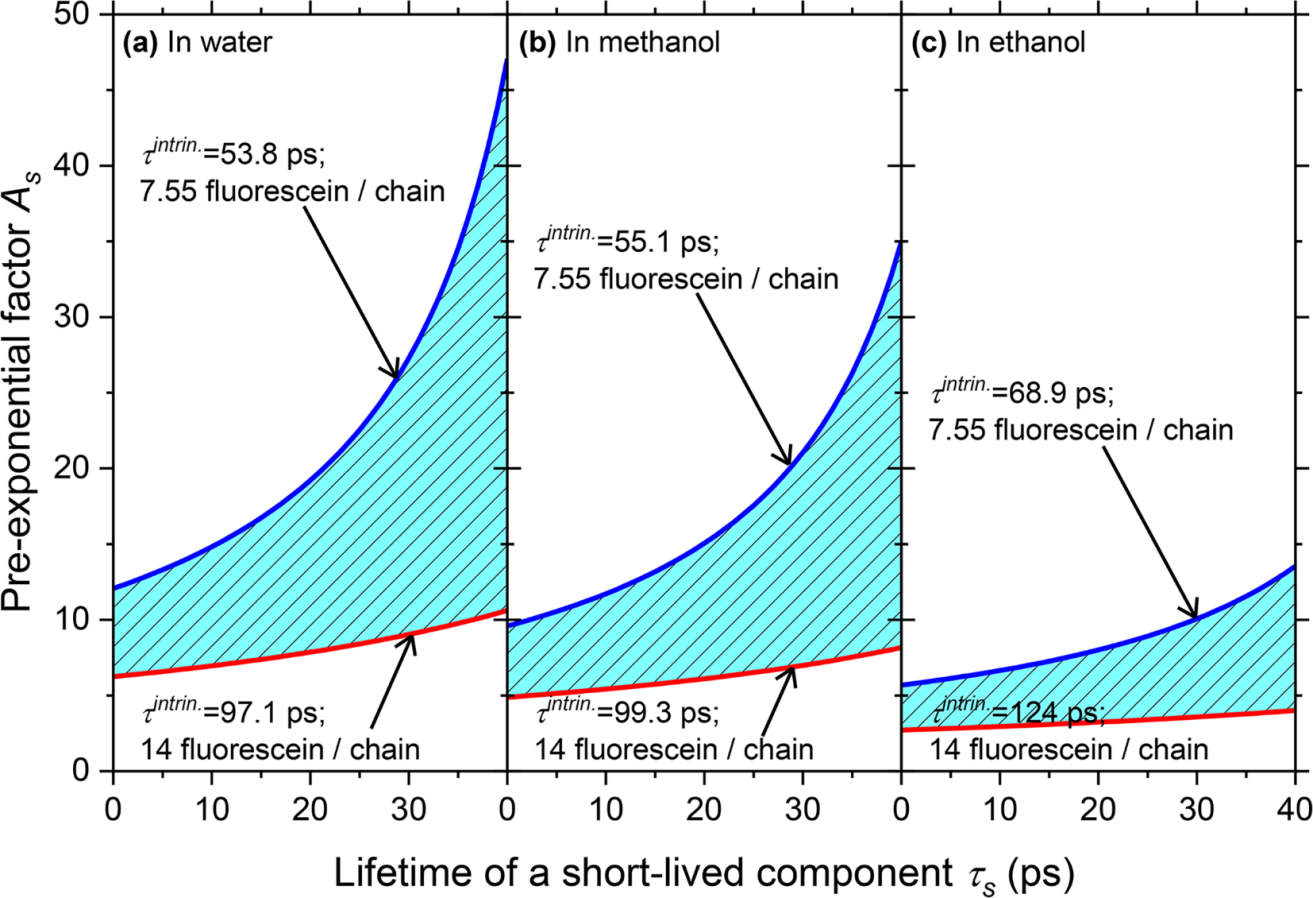
Relationships between the pre-exponential factor ${A}_{s}$ and lifetime ${\tau}_{s}$ of an anticipated extremely short-lived fluorescein component for time scales of 0.1‒40 ps, which is too fast to be detected, in (**a**) water, (**b**) methanol, and (**c**) ethanol. Hatched colored regions indicate the possible existence of an extremely fast component, which depends on the number of fluorescein moieties incorporated into a cellulose backbone.

Figure 4 shows the deduced pre-exponential factor ${A}_{s}$ of a short-lived component assuming that its possible ${\tau}_{s}$ values are undetectably short in the FLC solutions. Based on ${\phi}_{PL}$ and ${k}_{r}^{S.B.}$, ${\tau}^{intrin.}$is defined as ${\phi}_{PL}/{k}_{r}^{S.B.}$. When three components are considered, the decay profile $I\left(t\right)$ is represented by $I\left(t\right)={A}_{s}\text{exp}\left(-t/{\tau}_{s}\right)+{A}_{1}\text{exp}\left(-t/{\tau}_{1}\right)+{A}_{2}\text{exp}\left(-t/{\tau}_{2}\right)$. Thus, the average lifetime is described as $\left({A}_{s}{\tau}_{s}+{A}_{1}{\tau}_{1}+{A}_{2}{\tau}_{2}\right)/\left({A}_{s}+{A}_{1}+{A}_{2}\right)$, which corresponds to ${\tau}^{intrin.}$. Therefore,

1 $${\tau}^{intrin.}=\frac{{A}_{s}{\tau}_{s}+{A}_{1}{\tau}_{1}+{A}_{2}{\tau}_{2}}{{A}_{s}+{A}_{1}+{A}_{2}}=\frac{\left(\frac{{A}_{s}}{{A}_{1}+{A}_{2}}\right){\tau}_{s}+\left(\frac{{A}_{1}{\tau}_{1}+{A}_{2}{\tau}_{2}}{{A}_{1}+{A}_{2}}\right)}{\left(\frac{{A}_{s}}{{A}_{1}+{A}_{2}}\right)+1}\equiv\frac{A\:{\tau}_{s}+\:{\tau}_{ave}}{A\:+\:1}\:\:\: ,$$

where *A* is defined as ${A}_{s}/\left({A}_{1}+{A}_{2}\right)$; however, $A={A}_{s}$ when ${A}_{1}+{A}_{2}$ is unity. ${\tau}^{intrin.}$ can then be analyzed using the ${k}_{r}^{S.B.}$ values summarized in Table 2, the ranges of which are based on the ambiguity in quantifying the concentration ${C}_{FITC}^{FLC}$ of the fluorescein moieties in each FLC solution. Figure 4 covers the range of ${\tau}^{intrin.}$ for each FLC solution, and the pre-exponential factor ${A}_{s}=A$ with ${A}_{1}+{A}_{2}=1$ is obtained by assuming that ${\tau}_{s}$ ranges from 0.1 to 40 ps. The values of the pre-exponential factor in the graph correspond to the number of fluorescein moieties that are quenched when one fluorescein moiety fluoresces. Thus, although this number varies because of the ambiguity in how much fluorescein is incorporated into a single cellulose backbone, when one fluorescein moiety fluoresces, multiple fluorescein moieties are quasi-statically quenched owing to interactions with the cellulose backbone.

**Fig. 5:**
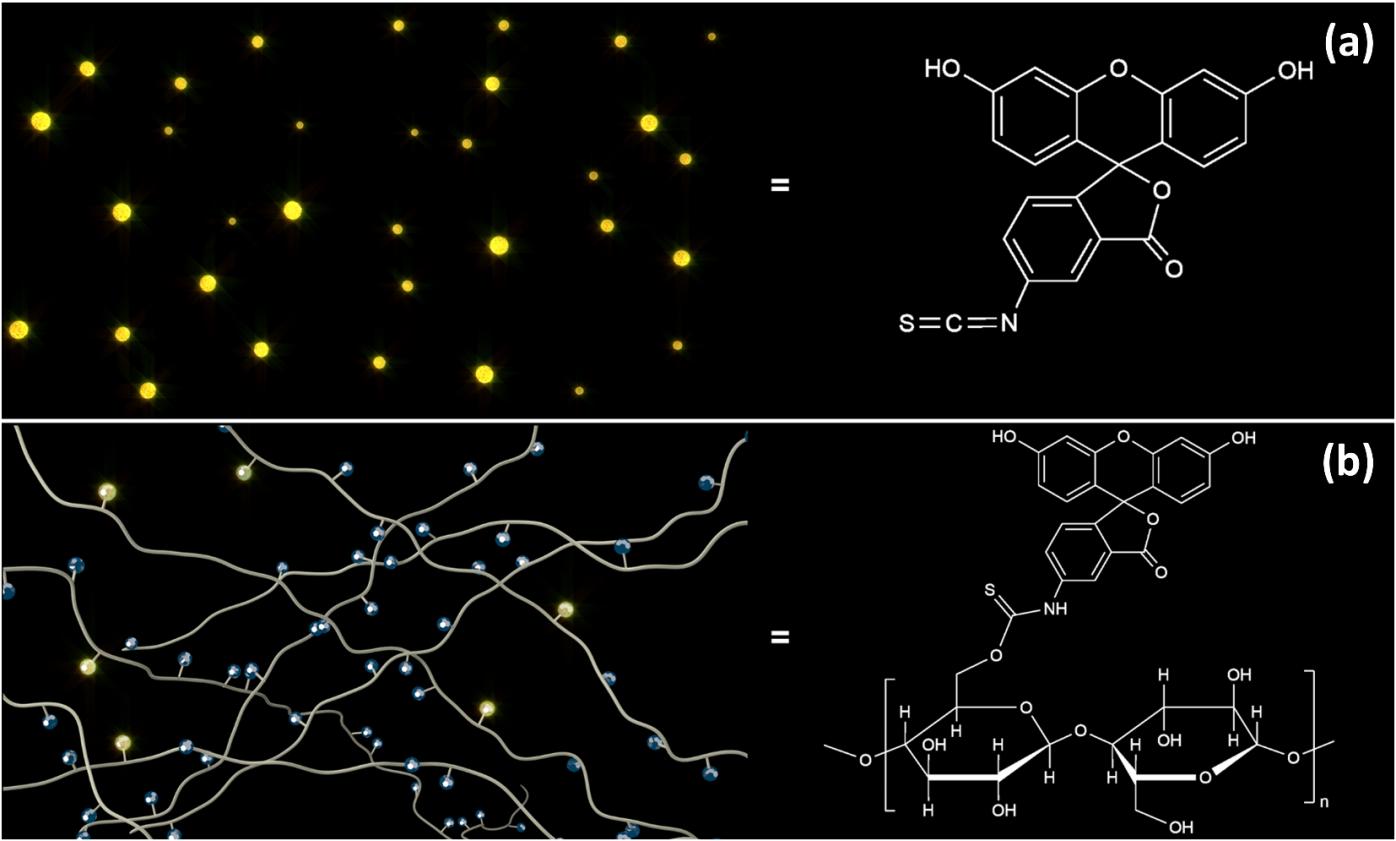
Illustrations comparing photoluminescence behavior (**a**) without and (**b**) with a cellulose backbone. In (**a**), FITC fluoresces more brightly and consistently (yellow spots), whereas in (**b**), most of the fluorescein moieties attached to the cellulose backbone are statically quenched (dark blue spots), and only a few of them can be interpreted as still fluorescing.

Figure 5 juxtaposes the photoluminescence behaviors of unbound FITC with those of the fluorescein moieties of FLC; in the latter, most of the fluorescein sites are quasi-statically quenched, with a few of sites still fluorescing. Based on this characterization, the number of unidentified short-lived fluorescein moieties per single fluorescing fluorescein moiety are estimated, as shown in Fig. 4. As the ${\tau}_{s}$ of the short-lived component becomes shorter, the number of quenched fluorescein sites decreases. This analysis also indicates that the efficiency of static quenching increases in the order of ethanol, methanol, and water. Further, the smaller the number of fluorescein moieties introduced into a cellulose backbone, the higher the probability of static quenching. These results cannot identify a specific interaction at the molecular level; however, hydroxyl groups on the cellulose backbone may play a role, as the backbone is mainly composed of these groups. Emphasis should be placed on the fact that the observed fluorescence is not caused by an impurity but originates from fluorescein moieties in the cellulose backbone, as the excitation spectra (dashed line, Fig. 2(b)) acquired multiple times from the FLC solutions are the same as their absorption spectra (Fig. 2(a)). This atypical type of static quenching may be referred to as quasi-static quenching, as the degree of quenching is incomplete, thus allowing some PL; this incomplete quenching can be attributed to fluorescent fluorescein moieties and components that are nearly quenched very quickly. Therefore, ${\tau}_{s}$ becomes too short to be detected, which was indeed the case with our results. Notably, the analysis in Fig. 4 suggests that as the number of fluorescein moieties incorporated into the cellulose backbone decreases, the probability increases that fluorescein is quenched by the cellulose backbone.

## Conclusion

We observed that the fluorescein moieties were quasi-statically quenched by the cellulose backbone, probably because of the weak interactions between the former and the latter; the quantum yields ${\phi}_{PL}$ of the FLC solutions were significantly reduced by a factor of ~ 30 with respect to those of the FITC solutions. Superficially, the decrease in ${\phi}_{PL}$ of the FLC solutions could be attributed to an anomalous decrease in the experimentally obtained rate constants of radiative decay ${k}_{r}$; however, this is not entirely the correct physical understanding. The rate constants of radiative decay calculated using the Strickler‒Berg formula ${k}_{r}^{S.B.}$ helped deduce the intrinsic rate constant of nonradiative decay ${k}_{nr}^{intrin.}$, which became enormous, as is the case with typical static quenching. The rate of nonradiative decay described herein is the effective rate of nonradiative decay, which includes the formation of a stable non-emissive complex between the fluorophore and the quencher, or the case in which the former and the latter are close enough to each other that quenching occurs. Dividing the lifetimes ${\tau^{intrin.}}$ that are obtained from ${k}_{r}^{S.B.}$ and ${k}_{nr}^{intrin.}$ into the experimentally observed ${\tau}_{i}$ and short-lived components ${\tau}_{s}$ allowed us to conclude that a large portion of the fluorescein moieties are statically quenched by interactions with the cellulose backbone, as shown in Fig. 5. Further, the number of quenched fluorescein sites is quantified as a function of the amount of FITC incorporated into the cellulose backbone.

In this study, fluorescein was used as a representative dye to observe whether the cellulose backbone influences the PL of dyes. The observed quenching is meaningful as it implies that the photophysics of dyes should not be ignored when introducing dyes into the cellulose backbone. Although the observed quenching can be plausibly interpreted, further efforts should be devoted to directly detecting the fast, imperfectly quenched luminescence component and revealing a detailed mechanism to explain how the cellulose backbone can quench the PL of dyes at the molecular level.

## Methods

### Materials and preparation

FLC was purchased from Creative PEGWorks with a specification of 5.0 mol% fluorescein-labeled cellulose, which represents the degree of substitution, i.e., the ratio of the number of fluorescein moieties to cellulose subunits. Given a molecular weight (MW) of 324 for a cellulose subunit (C_12_H_20_O_10_)_n_, approximately 14 fluorescein moieties were therefore introduced into the polymer chain of cellulose with a MW of 90,000 g/mol; however, the number of fluorescein moieties introduced into a single chain was ambiguous, as is discussed in “Quantifying the fluorescein moieties per chain.” Note that the FLC concentration in solution can be defined by the concentration ${C}_{FITC}^{FLC}$ of fluorescein moieties, whereas ${C}_{chain}^{FLC}$ denotes the concentration of FLC chains. FITC (Product#: F7250, Sigma-Aldrich) was used without further purification, and its concentration in solution is represented by ${C}_{FITC}$.

To prepare solutions of FITC (MW: 389.38) and FLC (MW: 92,939.82‒95,451.32, which corresponds to 7.55‒14.0 fluorescein moieties per single cellulose chain), predefined quantities of 7.79 × 10^−3^ g and 9.00 × 10^−3^ g were weighed and placed in 20 mL and 10 mL volumetric flasks, respectively. Dimethyl sulfoxide (DMSO) was added to each flask, and each mixture was stirred with a magnetic stirrer at 200 rpm for 24 h to obtain stock solutions in DMSO with ${C}_{FITC}=\:$1.00 mM FITC and ${C}_{chain}^{FLC}=\:$9.43‒9.68 μM FLC. A certain quantity of the stock solution was appropriately diluted using deionized water, ethanol, or methanol to obtain a series of samples.

## Characterization

Steady-state UV‒Vis absorption spectra were recorded using a JASCO V-780 spectrophotometer, and emission and excitation spectra were acquired on a JASCO FP-6300 spectrofluorometer. Fluorescence decay profiles were recorded using time-correlated single photon counting, as described elsewhere^75,76^. The quantum yield ${\phi}_{PL}$ is equivalent to the ratio of the radiative transition rate constant to the sum of all rate constants involved in deactivating the excited state, and it can be described as ${\phi}_{PL}={k}_{r}/({k}_{r}+{k}_{nr})=1/(1+{k}_{nr}/{k}_{r})={k}_{r}\tau$, where ${k}_{r}$, ${k}_{nr}$, and ${\tau}$ are the radiative and nonradiative decay constants and the fluorescence lifetime, respectively. Therefore, all decay constants can be determined by experimentally acquiring ${\phi}_{PL}$ and ${\tau}$.

Complementary PL characteristics decomposed into the fast and slow components of the PL decay profiles in the FLC solutions are summarized in the Supplementary Information (PDF).

## Electronic supplementary material

Below is the link to the electronic supplementary material.

Supplementary Material 1

## Data Availability

The datasets generated during and/or analyzed during the current study are available from the corresponding author on reasonable request.
