# Supplementary material for: Characterizing the photoluminescence of fluorescein-labeled cellulose in aqueous and alcohol solutions: influence of the cellulose backbone
Source: Sci Rep. 2024 Oct 31;14:26223. doi: 10.1038/s41598-024-72773-6 (PMC11528010; doi:10.1038/s41598-024-72773-6)
Supplement: Supplementary file 1 — Supplementary Material 1 [file 41598_2024_72773_MOESM1_ESM.pdf]

Supplementary Information

Characterizing the photoluminescence of fluorescein-labeled cellulose in aqueous and alcohol solutions: influence of the cellulose backbone

Chi-Yang Yen[1], *Shailesh Rana*[2], *Kamlesh Awasthi*[2], *Nobuhiro Ohta*[2,*], *Masahito Oh-e*[1,*]

[1] Institute of Photonics Technologies, Department of Electrical Engineering,
National Tsing Hua University, 101 Sec. 2 Kuang-Fu Road, Hsinchu 300044, Taiwan

[2] Department of Applied Chemistry and Institute of Molecular Science, and Center for Emergent Functional Matter Science, National Yang Ming Chiao Tung University, 1001 Ta-Hsueh Road, Hsinchu 300093, Taiwan

* Corresponding authors

This Supplementary Information includes the complementary photoluminescence (PL) properties of fluorescein-labeled cellulose (FLC) in water, methanol, and ethanol.

Using the fast τ_1 and slow τ_2 components enabled deducing their respective quantum yields as well as the rate constants of radiative and nonradiative decay for each component, as summarized in Tables S1 and S2. Notably, the rate constants k_r^{fast} and k_r^{slow} of radiative decay, which were obtained from the τ_1 and τ_2 components of FLC, are still about one order of magnitude lower and even lower than those of the single decay component in the fluorescein isothiocyanate (FITC) counterpart solutions. This suggests that the lifetime of the PL component that exhibits static quenching is still much shorter (and hence undetectable) than that of the fast-decaying component observed in the PL decay profiles. The data from the decomposition are consistent with the conclusion obtained by comparing the radiative decay rate constants determined using the total fluorescence quantum yield ϕ_{PL} and the average lifetime τ_{avg} in the main text.

Supplementary Table S1. Quantum yields ϕ_{PL}^{fast} and ϕ_{PL}^{slow} of the fast τ_1 and slow τ_2 components.

Solute	Solvent	ϕ_{PL}	$\frac{A_1\tau_1}{A_1\tau_1 + A_2\tau_2}$	$\frac{A_2\tau_2}{A_1\tau_1 + A_2\tau_2}$	ϕ_{PL}^{fast} (τ_1, ns)	ϕ_{PL}^{slow} (τ_2, ns)
FLC	Water	0.014	0.097	0.903	0.0014 (0.087)	0.0126 (2.79)
	Methanol	0.014	0.169	0.831	0.0024 (0.115)	0.0113 (2.69)
	Ethanol	0.018	0.245	0.755	0.0044 (0.127)	0.0136 (2.45)

Note: The fluorescence was monitored at λ = 520 nm. $A_1\tau_1/(A_1\tau_1 + A_2\tau_2)$ and $A_2\tau_2/(A_1\tau_1 + A_2\tau_2)$ are the intensity ratios of the rapidly and slowly decaying components, respectively. As the PL of the FITC solutions exhibits a mono-exponential decay profile, decomposing ϕ_{PL} is not required.

Supplementary Table S2. Rate constants of radiative (k_r^{fast} and k_r^{slow}) and nonradiative (k_{nr}^{fast} and k_{nr}^{slow}) decay of the fast τ_1 and slow τ_2 components.

Solute	Solvent	ϕ_{PL}^{fast} (ϕ_{PL}^{slow})	k_r^{fast} (k_r^{slow}) $\times 10^8$ / s^{-1}	k_{nr}^{fast} (k_{nr}^{slow}) $\times 10^9$ / s^{-1}	$k_r^{S.B.}$ $\times 10^8$ / s^{-1}
FLC	Water	0.0014 (0.0126)	0.157 (0.045)	11.48 (0.354)	1.40–2.53*
	Methanol	0.0024 (0.0116)	0.206 (0.045)	8.68 (0.367)	1.36–2.45*
	Ethanol	0.0044 (0.0136)	0.347 (0.055)	7.84 (0.402)	1.41–2.54*

Note: k_r^{fast} (k_r^{slow}) and k_{nr}^{fast} (k_{nr}^{slow}) were determined from ϕ_{PL}^{fast}/τ_1 $(\phi_{PL}^{slow}/\tau_2)$ and $1/\tau_1 - k_r^{fast}$ $(1/\tau_2 - k_r^{slow})$, respectively. $k_r^{S.B.}$ is the rate constant of radiative decay calculated using the Strickler–Berg formula. *See the section "Quantifying the fluorescein moieties per chain" in the main text for the estimation of these values.
